# Cardiogenic Shock due to Kounis Syndrome following Cobra Bite

**DOI:** 10.1155/2019/5185716

**Published:** 2019-08-04

**Authors:** W. D. D. Priyankara, E. M. Manoj, A. Gunapala, A. G. R. M. A. Ranaweera, K. S. Vithanage, M. Sivasubramanium, E. Snajeeva

**Affiliations:** ^1^National Hospital of Sri Lanka, Sri Lanka; ^2^Critical Care Medicine National Hospital of Sri Lanka, Sri Lanka

## Abstract

Kounis syndrome is associated with mast cell activation resulting in acute coronary syndrome secondary to an allergic insult. Various drugs such as antibiotics, analgesics, and environmental exposures such as bee, wasp sting, and poison ivy are known to induce Kounis syndrome. A 68-year-old man admitted with a cobra bite on both hands to emergency care unit and sustained cardiorespiratory arrest. Electrocardiogram, taken 6 hours after the cardiac arrest, showed ST elevations in leads V_2_ to V_5_ suggestive of anterior ST elevation myocardial Infarction (STEMI). Serum Troponin was 10 ng/ml (control= <0.5). Serum IgE levels were significantly high (19155IU/ml, baseline 100). 2-Dimensional echocardiogram showed anterior and apical-septal hypokinesia with left ventricular ejection fraction of 30-35%. Coronary angiogram was normal. He remained hypotensive requiring inotropic and vasopressor support during ICU stay. This was a case of Kounis syndrome leading to cardiogenic shock secondary to Cobra (naja naja) bite. This is the only reported case of cobra bite causing Kounis syndrome and cardiogenic shock. Identification of the cause of myocardial infarction in snake envenomation is useful in the management as some of the drugs like adrenaline, morphine, and beta blockers may worsen the clinical syndrome if it is due to Kounis syndrome.

## 1. Introduction

Kounis syndrome (KS) which is also known as allergic angina syndrome is a hypersensitivity reaction to an allergic insult. This is associated with mast cell activation resulting in acute coronary syndrome (ACS). KS has been reported to associate with viper bites. However, KS associated with cobra bite has never been reported. We report a case of cardiogenic shock due to KS following cobra (*Naja naja*) bite.

## 2. Case Report

**Figure 1:**
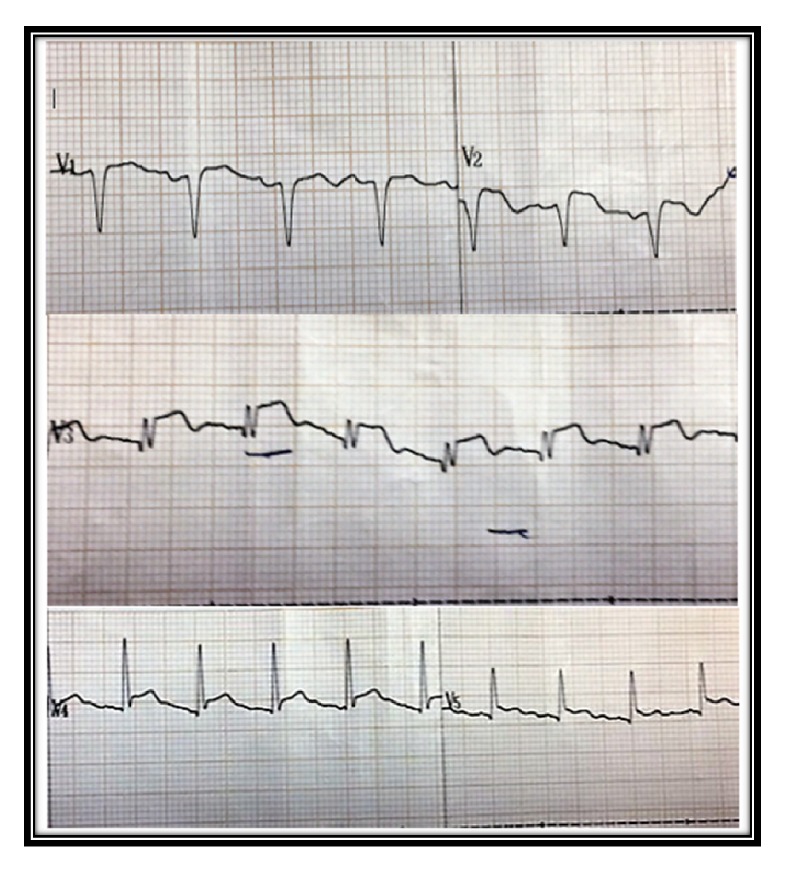
ECG of the patient after the cardiac arrest showing ST elevation in anterior leads.

**Figure 2:**
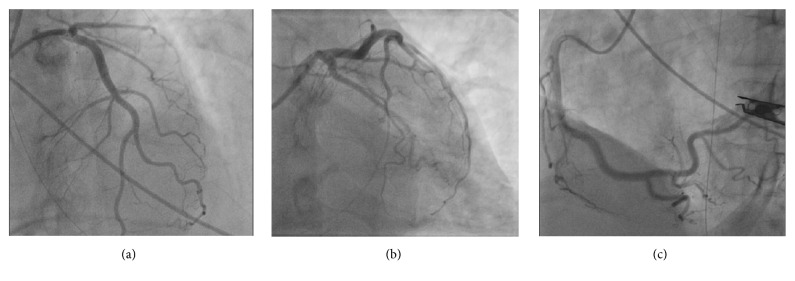
Coronary angiogram showing minor coronary artery disease without culprit lesions. (a) Left anterior descending artery, (b) left circumflex artery, and (c) right coronary artery.

A 68-year-old manual labourer was admitted to the local hospital following a cobra bite on both hands and was transferred to a tertiary care hospital for specialized care. On admission to the emergency care unit (ETU), he sustained cardiorespiratory arrest. He was quickly resuscitated and transferred to our intensive care unit for further care. Meanwhile, he was treated with 100 ml of Indian poly-specific antivenom serum immediately following return of spontaneous circulation (ROSC). He received one dose of intravenous hydrocortisone of 200 mg at the ETU. However, he did not continue on steroids. He developed supraventricular tachycardia immediately after ROSC and was treated with intravenous verapamil to beneficial effect. His Electrocardiogram (ECG) immediately after the cardiac arrest was normal. However, ECG taken 6 hours after the cardiac arrest showed ST elevations in leads V_2_ to V_5_ suggestive of anterior ST elevation myocardial Infarction (STEMI) (Figure 1). Serum Troponin was 10 ng/ml (control= <0.5) at 24 hours. Serum IgE levels measured on day 2 were significantly high (19155IU/ml, baseline 100). However, the eosinophil count was 0.02 × 10^3^/*μ*L. He remained hypotensive requiring inotropic and vasopressor support (noradrenaline 0.6*μ*g/kg/min, dobutamine 5 *μ*g/kg/min) during 1st 72 hours after cardiac arrest. 2-Dimensional echocardiogram (2D ECHO) performed on day 1 in the ICU showed anterior and apical-septal hypokinesia with left ventricular ejection fraction of 30-35%. On the same day coronary angiogram revealed minor coronary artery disease without any culprit lesion compatible with anterior ischaemia (Figure 2). His cardiovascular supports and respiratory supports were weaned off over 48 hours. Hand wound was treated with intravenous antibiotics and surgical debridement. After 5 days of ICU stay he was transferred to a medical ward. His ECG normalised and the repeated ECHO in one week was normal.

## 3. Discussion

KS was first described by Kounis and Zavras in 1991 as a clinical syndrome characterised by angina pectoris and allergic reaction to an allergic insult [1]. KS is thought to be mediated by histamine and leukotrienes which leads to coronary artery spasm [2]. Three subtypes of KS have been described. Type 1 occurs in patients with normal coronary arteries and type 2 occurs in patients with preexisting atheromatous coronary artery disease. Type 3 will lead to stent thrombosis. All three conditions can lead to angina and myocardial infarction [3].

There are several identified causative factors which can induce KS. Various drugs such as antibiotics, analgesics, and environmental exposures such as bee, wasp sting, and poison ivy are known to induce KS [3]. Antibiotics following insects' bites are reported to be the most common triggers of KS [3]. Snake venom can induce allergic or anaphylactic reactions due to various substances like toxic peptides, phospholipase A2, and various other proteins present in their venom [4]. Furthermore, there are several case reports of viper bites leading to KS [5, 6].

Ruth and colleagues reported hypersensitivity to spitting airborne cobra venom among cobra handlers. It further highlighted that snake venom could be a source of anaphylaxis in sensitised individuals [7]. However, no case has been reported on KS secondary to cobra bite.

Cardiac injury following snake bite is well documented. However, the exact mechanism still remains unclear. Hypovolemia, direct cardiotoxicity, hypercoagulability, and vasospasm are reported as possible mechanisms [4]. Myocardial infarction is more reported mainly in viper bites than in cobra bites [8].

In our patient the initial dilemma was whether the cardiogenic shock is related to snake bite or not. Coronary angiogram showed only mild disease ruling out ACS due to atherosclerotic plaque rupture or thrombosis. 2D ECHO showed anterior regional wall motion abnormalities compatible with anterior ST segment elevations in the ECG. Even though the direct cardiac toxicity would be difficult to exclude, it would have caused global hypokinesia compared to regional one. Therefore, these two factors were more in favour of vasospasm than acute atherosclerotic and direct myocardial injury following cobra envenomation. Furthermore, highly raised IgE supports the presence of acute allergic reaction. All these would be characteristic of type 1 KS. Furthermore, we did not have facilities to perform serum tryptase levels.

Steroids may alleviate biphasic anaphylactic reaction and arterial hyperactivity [9, 10]. Epinephrine should be used with caution in KS as it may have detrimental effects on myocardial perfusion and may induce arrhythmias [9]. Furthermore, morphine used for angina may worsen allergic reaction in KS because mast cell degranulation and beta blocker may worsen vasospasm. Therefore, identification of the cause of myocardial infarction in snake envenomation is useful in the management.

Literature shows that serious complications such as cardiogenic shock, cardiac arrest, and death are rare due to KS [11]. However, our patient suffered cardiac arrest and cardiogenic shock.

## 4. Conclusion

This case highlights a case of Kounis syndrome due to cobra bite leading to cardiogenic shock. This is the only reported case of cobra bite causing Kounis syndrome and cardiogenic shock. Identification of the reason for the myocardial infraction in cobra bites is important as the management would differ as the routine management of patients with atherosclerotic ACS is not applicable in every situation. Furthermore, the prognosis of KS is better than atherosclerotic ACS.
